# Prediction of early C-reactive protein levels after non-cardiac surgery under general anesthesia

**DOI:** 10.1371/journal.pone.0226032

**Published:** 2019-12-02

**Authors:** Shiroh Nakamoto, Munetaka Hirose

**Affiliations:** Department of Anesthesiology and Pain Medicine, Hyogo College of Medicine, Hyogo, Japan; Cleveland Clinic, UNITED STATES

## Abstract

**Background:**

Early detection of postoperative increase in C-reactive protein (CRP) correlates with postoperative complications. The present study examined the association between preoperative / intraoperative factors and postoperative CRP levels, with development and validation of a prediction model of early postoperative CRP level, for prophylactic management of postoperative complications in patients undergoing surgery under general anesthesia.

**Material and methods:**

Multivariate regression analysis was retrospectively performed to determine the independent factor of CRP levels on postoperative day (POD) 1 and to develop a prediction model. Validation of the prediction model was prospectively performed. Data from 316 adult patients on perioperative variables were retrospectively obtained in a training cohort in patients undergoing elective non-cardiac surgery. In a validation cohort, 88 patients undergoing mastectomy and 68 patients undergoing laparoscopic colon surgery were prospectively utilized to evaluate the value of the prediction model. Major complications after surgery were defined as the Clavien-Dindo grade IIIa or greater.

**Results:**

Duration of surgery, mean nociceptive response (NR) during surgery as intraoperative nociception level, and preoperative CRP level were selected to set up the prediction model of CRP level on POD1 (*P* < 0.0001). In the validation cohort, the predicted CRP levels on POD1 significantly correlated with the measured CRP after mastectomy (*P* < 0.0001) and laparoscopic colon surgery (*P* = 0.0001). Receiver-operating characteristic curve analysis showed that the predicted CRP levels on POD1 was significantly associated with major complications after mastectomy (*P* = 0.0259) and laparoscopic colon surgery (*P* = 0.0049). The measured and predicted CRP levels significantly increased in the order of severity of postoperative complications (*P* < 0.01).

**Conclusion:**

Increases in duration of surgery, intraoperative nociceptive level and preoperative CRP level were selected to predict early increases in CRP level after non-cardiac surgery under general anesthesia. Predicted CRP levels on POD1 were likely associated with severity of postoperative complications.

## Introduction

Early diagnosis and treatment of postoperative complications is crucial for suppression of the associated morbidity and mortality. C-reactive protein (CRP) is an acute phase reactant, which increases postoperatively in response to inflammation, intraoperative tissue damage or blood loss. Recently, CRP is increasingly being studied as an early marker of postoperative complications, with early detection of an increase in CRP levels on postoperative days (POD) 1–3 reportedly being predictive of the occurrence of complications after non-cardiac surgery [1–3]. Since the preoperative and intraoperative factors associated with postoperative CRP changes would be correlated with postoperative complications, perioperative managements for controlling those preoperative and intraoperative factors are potentially significant in the prophylactic management of postoperative complications. Those factors, however, have not been evaluated for the association with postoperative CRP levels after surgery. To reveal the relationship between perioperative risk factors and early postoperative C-reactive protein levels, we developed and validated a prediction model for CRP levels on POD1 in training and validation cohorts in patients undergoing non-cardiac surgery in the present study.

## Methods

This study was approved by the Ethics Committee of Hyogo College of Medicine (Ethical Committee number 3138; Chairperson—Koichi Noguchi). The requirement for written informed consent was waived by the institutional ethics committee in a retrospective cohort. In a prospective cohort, informed consent was obtained in the form of opt-out on the web-site. This study was conducted in accordance with the principles of the Declaration of Helsinki.

In a training cohort to develop the prediction model, patients who underwent elective mastectomy, spine surgery, laparoscopic surgery, open abdominal surgery, or thoracic surgery, were retrospectively selected from May 2018 to August 2018, as CRP levels on POD1 were routinely measured after those surgeries. As American Society of Anesthesiologists-physical status (ASA-PS) was reported to associate strongly with postoperative complications [4, 5], we determined eligibility criteria for the training cohort as the following: age over 20 years and ASA-PS I–II.

In a validation cohort to verify the value of the prediction model, consecutive patients who underwent elective mastectomy or laparoscopic colon surgery were prospectively selected from September 2018 to March 2019. As preoperative CRP levels were also reported to associate with postoperative complications [6, 7], we determined eligibility criteria for the validation cohort as the following: age over 20 years, ASA-PS I–II, and preoperative CRP level <0.3 mg·dL^-1^.

### Perioperative management

None of the patients received premedication. General anesthesia was induced with propofol, fentanyl and rocuronium, followed by insertion of a tracheal tube or supraglotic airway, and was maintained with sevoflurane / desflurane, fentanyl, rocuronium and a continuous infusion of remifentanil. The doses of remifentanil and fentanyl were adjusted to maintain mean blood pressure within a range of ± 20% of the pre-anesthesia level. Additional regional anesthesia was determined by anesthesiologists in charge. Bispectral index was maintained between 40 and 60 by adjusting the concentration of sevoflurane / desflurane. Rocuronium bromide was used for muscle relaxation during surgery, as needed. After surgery, continuous administration of intravenous fentanyl with 25–50μg.hr^-1^, intravenous flurbiprofen axetil, or acetaminophen was performed until POD 1–2 for postoperative analgesia.

### Data collection

Information on serum concentrations of CRP before and one day after surgery, and complications occurring during hospitalization within 30 days after surgery were obtained from our institutional medical records. The normal range for CRP at our institution is below 0.3 mg·dL^-1^. Postoperative complications were graded according to the extended Clavien-Dindo classification, which includes seven grades; grade I (any deviation from the normal postoperative course), grade II (normal course altered), grade IIIa (complications that require interventions performed under local anesthesia) or grade IIIb (complications that require interventions performed under general or epidural anesthesia), grade IVa (life-threatening complications with single organ dysfunction), grade IVb (life-threatening complications with multi-organ dysfunction), and grade V (death) [8]. Major complication was defined as the extended Clavien-Dindo grade IIIa or greater.

Real-time nociception during surgery was calculated using Nociceptive Response (NR), which represents objective indices of surgical invasiveness [9]. NR is a hemodynamic equation including heart rate (HR), systolic blood pressure (SBP), and perfusion index (PI), as follows [9]:

$$NR=-1+\frac{2}{1+e^{-0.01HR-0.02SBP+0.17PI}}.$$

Intraoperative nociception, which reportedly correlates with major postoperative complications [10], was evaluated using the averaged value of NR from the start to end of surgery (mean NR) [10, 11]. After we installed the equation of NR on our institutional anesthesia information managing system (ORSYS, PHILIPS Japan, Tokyo, Japan) in May 2018, mean NR was routinely monitored, being obtained using the data-search software (Vi-Pros, Dowell, Sapporo, Japan).

### Statistics

All statistical testing was two-sided with a significance level of 5% and was performed using JMS Pro version 13.1.0 (SAS Institute Inc. Cary, NC, United States). The Kruskal-Wallis test and the Wilcoxon test were used to compare variables between training and validation cohorts. To investigate the association between CRP levels on POD1 and preoperative/intraoperative variables (age, sex, BMI, ASA-PS, duration of operation, mean NR, and preoperative CRP level), we performed multiple linear regression analyses in the training cohort, and then developed the prediction model for CRP level on POD1. The prediction model was applied on the validation cohort to evaluate its value using linear regression analysis between measured and predicted CRP levels. The prediction of major complications using this model was evaluated in the validation cohort using a receiver-operating characteristic (ROC) curve analysis.

## Results

### Development of prediction model

A total of 316 patients undergoing non-cardiac surgery in the training cohort were assessed for eligibility (Table 1). The number of operations selected were 39 for mastectomy, 59 for spine surgery, 95 for laparoscopic surgery, 66 for open abdominal surgery, and 57 for thoracic surgery. We performed multivariate linear regression analysis to explore predictive factors for the increase in postoperative CRP levels, which showed that CRP levels on POD1 (mg•dL^-1^) were significantly associated with the duration of surgery (min), mean NR value during surgery, and preoperative CRP level (mg•dL^-1^) (Table 2). The prediction model was developed as follows:

$$\begin{array}{l} Predicted\;CRP\;levels\;on\;POD1\\ \;=-4.38+0.0058Duration\;of\;surgery+6.44Mean\;NR\\ \;+0.44Preoperative\;CRP\;level \end{array}$$

**Table 1:** Comparison of patient demographics between training and validation cohorts.

Variables	Training cohort (n = 316)	Validation cohort	
		Mastectomy (n = 88)	Laparoscopic colon surgery (n = 68)
Age (years)	63 ± 14	56 ± 13**	58 ± 18*
Sex (M/F)	155/161	1/87**	42/26^#^
BMI	22.7 ± 3.5	23.0 ± 3.3	22.0 ± 3.5
ASA-PS I/II	48/267	29/59**	11/57^##^
Duration of surgery (min)	210 ± 136	179 ± 122*	249 ± 126**^##^
Mean NR	0.82 ± 0.05	0.78 ± 0.05**	0.81 ± 0.05^##^
Preoperative CRP level (mg•dL^-1^)	0.26 ± 0.90	0.07 ± 0.07**	0.09 ± 0.08^#^
CRP level on POD1 (mg•dL^-1^)	2.27 ± 2.62	0.94 ± 1.35**	3.39 ± 2.19**^##^

Data are presented as mean ± SD. ASA-PS: American Society of Anesthesiologists physical status, BMI: body mass index, CRP: C-reactive protein, NR: nociceptive response, POD: postoperative day.

*P<0.05 and

**P<0.01 vs. Training cohort.

^#^*P*<0.05 and

^##^*P*<0.01 vs. Mastectomy.

**Table 2:** Multivariate linear regression analysis of the association between CRP levels on POD1 and preoperative and intraoperative variables in training cohort.

Variables	β	*P*
Age (years)	-0.003	0.9640
Sex (M/F)	-0.077	0.1538
BMI	-0.043	0.4209
ASA-PS (I/II)	0.058	0.2947
Duration of surgery (min)	0.297	<0.0001**
Mean NR	0.120	0.0347*
Preoperative CRP level (mg•dL^-1^)	0.139	0.0091**

Data are presented as standardized beta coefficient (β). ASA-PS: American Society of Anesthesiologists physical status, BMI: body mass index, CRP: C-reactive protein, NR: nociceptive response, POD: postoperative day.

*P<0.05 and

**P<0.01 are significant.

### Validation of prediction model

**Fig 1:**
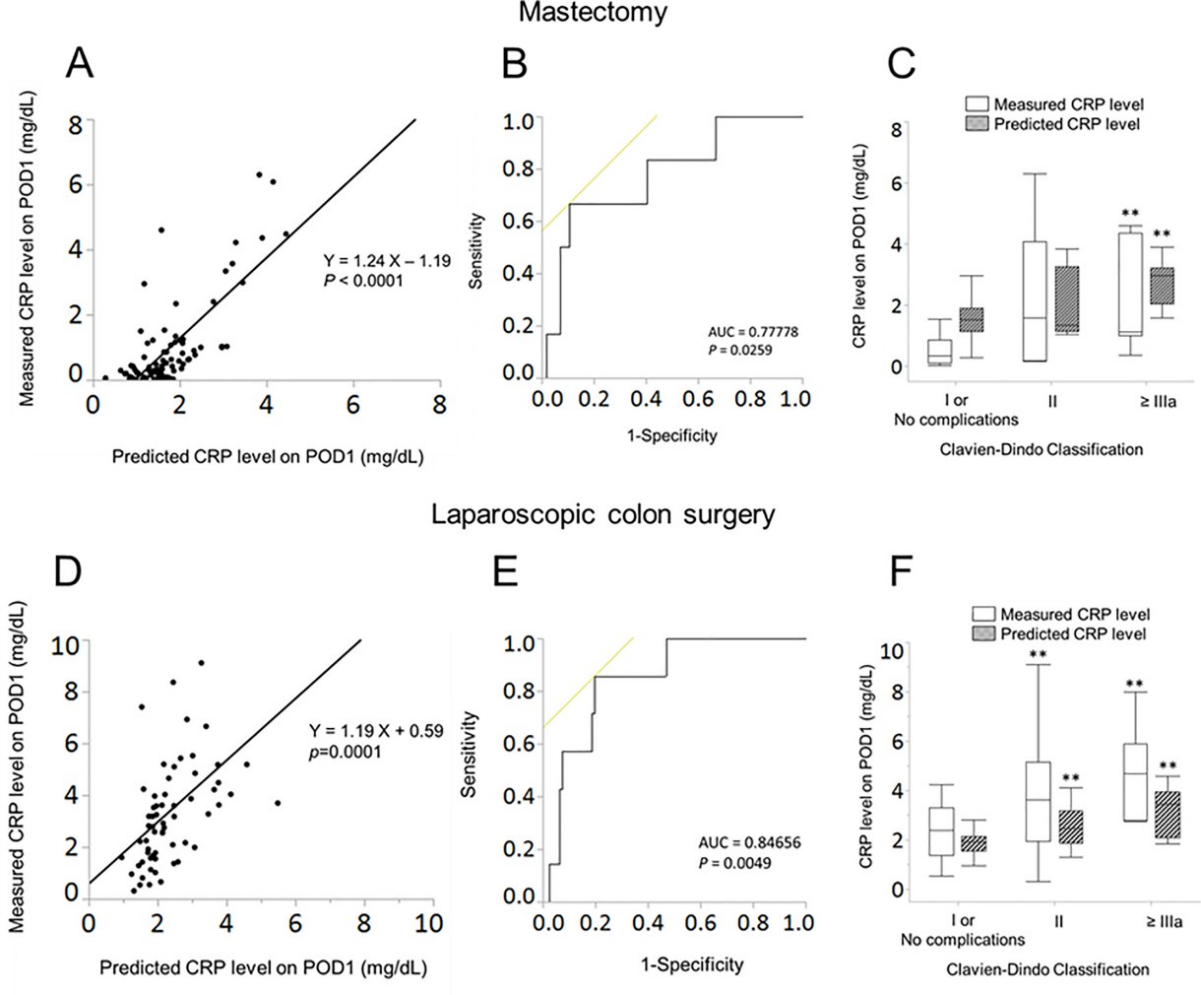
Relationships between predicted and measured CRP levels on POD1 and postoperative complications. Scatter-points represent actual data for 88 patients undergoing mastectomy (A) and for 68 patients undergoing laparoscopic colon surgery (D). Receiver operating characteristic curves for major complications (the Clavien-Dindo grade IIIa or greater) after mastectomy (B) and laparoscopic colon surgery (E) with predicted C-reactive protein levels on postoperative day 1 in the validation cohort. Box and whisker plots show associations between postoperative complications, measured CRP levels, and predicted CRP levels after mastectomy (C) and laparoscopic colon surgery (F). Postoperative complications were graded as: no complication or Clavien-Dindo grade I, Clavien-Dindo grade II, and Clavien-Dindo grade IIIa or greater. Statistically significant levels were considered as **P < 0.01 vs. no complications or Clavien-Dindo grade I. CRP: C-reactive protein, POD: postoperative day.

A total of 88 patients undergoing mastectomy and 68 patients undergoing laparoscopic colon surgery in the validation cohorts were assessed for eligibility (Table 1). Table 1 shows significant differences of perioperative variables between training and validation cohorts. Predicted CRP levels on POD1 showed a significant correlation with measured CRP levels on POD1 in two validation cohorts (Fig 1). Major complications occurred in 18 patients (5.7%) in the training cohort, 7 patients (8.0%) undergoing mastectomy, and 6 patients (8.8%) undergoing laparoscopic colon surgery. Major complications were surgical site infection (n = 3), bleeding (n = 3), and tissue ischemia (n = 1) after mastectomy, and surgical site infection (n = 2), ileus (n = 2), and others (n = 2) after laparoscopic colon surgery. Fig 1 shows ROC curves for major complications with predicted CRP levels on POD1, indicating that optimal cut-off value of the predicted CRP level on POD1 was 2.0 mg·dL-1 with a sensitivity of 0.86 and specificity of 0.80 after mastectomy, and was 3.2 mg·dL-1 with a sensitivity of 0.67 and specificity of 0.89 after laparoscopic colon surgery. The area under the curve (AUC) obtained in the ROC curve was 0.78 for the predicted CRP level after mastectomy and was 0.85 for the predicted CRP level after laparoscopic colon surgery (Fig 1). Both the measured and predicted CRP levels were significantly higher in patients with the Clavien-Dindo grade II after laparoscopic surgery and in patients with the grade IIIa or greater after mastectomy and laparoscopic surgery than those in patients with the Clavien-Dindo grade I or no complications (Fig 1).

## Discussion

Longer surgical duration, higher mean intraoperative NR value, and higher preoperative CRP level significantly correlated with the higher CRP value on POD1 in the present study. Severity of postoperative complications were significantly associated with both the measured and predicted CRP levels on POD1after mastectomy and laparoscopic colon surgery. In addition to duration of surgery or preoperative CRP level [7, 12–17], intraoperative nociception also has been reported to be a risk factor for postoperative complications [10].

Nociceptive stimulation during surgery increases intraoperative inflammation, tissue damage, and blood loss. It activates the autonomic nervous system and the hypothalamic-pituitary-adrenal axis, inducing potentially harmful outcomes such as cardiac dysfunction, vascular instability, or coagulopathy [18]. Several nociceptive indices using variables derived from autonomic responses indicate the degree of nociceptive stimulation under general anesthesia. The analgesia nociception index (ANI) is calculated from heart rate variability [19], and the surgical pleth index (SPI) is calculated using photoplethysmographic pulse wave amplitude and heart beat interval [20]. These previous indices, however, have not rated averaged nociceptive levels throughout surgery. On the other hand, the averaged values of NR, which is calculated using the variables of heart rate, systolic blood pressure, and perfusion index, represents the intraoperative nociceptive level in each surgery under general anesthesia [10, 11]. Mean NR is affected by surgical invasiveness and anesthetic managements, including analgesics, regional anesthesia, infusion or transfusion [10, 11]. Given that minimizing surgical trauma by performing laparoscopy instead of open surgery reportedly reduces early postoperative CRP levels [21–23], it seems plausible that the mean NR value was selected for the prediction of early postoperative CRP level in the present study.

Prevalence of major complications, which was defined as Clavien-Dindo grade ≥IIIa, reportedly ranged from 8.1 to 18.1% after laparoscopic gastrointestinal surgery [7, 24]. In the present study, the prevalence was 8.8% in patients undergoing laparoscopic colon surgery in the validation cohort. Since preoperative increase in CRP levels was selected to predict increase in postoperative CRP levels in the training cohort in the present study, we enrolled patients with ASA-PS I/II whose preoperative CRP levels were within the normal range to eliminate the confounding effect of preoperative CRP elevation in the validation cohort. The relatively low level of prevalence of major complications in the validation cohort, compared to the previous reports, might be caused by our selection criteria of patients without serious preoperative conditions or comorbidities. The effects of serious preoperative conditions or comorbidities, if any, might exceed the effects of duration of surgery, intraoperative nociceptive level, and preoperative CRP level on early CRP levels after non-cardiac surgery.

Perioperative managements reducing early postoperative CRP levels could prevent postoperative complications. Previously, reduction of surgical invasion [25–27], administration of non-steroidal anti-inflammatory drug [28], steroid [29], or β-blocker [30], and Enhanced Recovery after Surgery (ERAS) protocol [27, 31], were reported to reduce postoperative CRP levels. Although the effects of anesthetic managements on postoperative CRP levels are controversial so far [32, 33], combination with these perioperative managements could be expected to reduce postoperative CRP levels. Given that the prediction model for CRP levels on POD1 includes three prediction factors of duration of surgery, preoperative CRP levels, and intraoperative nociception being affected by surgical invasiveness and anesthetic managements, the incidence of postoperative complications could be reduced by an appropriate use of analgesics, β-blocker, or regional anesthesia during anesthesia, in addition to preoperative managements to suppress preoperative CRP levels and intraoperative surgical managements to reduce surgical invasiveness and duration of surgery. Further investigations are, however, needed to confirm this hypothesis.

A limitation of this study is that predicted model for CRP levels on POD1 was validated only in patients undergoing mastectomy or laparoscopic colon surgery. Further investigations are needed to validate this model more precisely in patients undergoing other surgeries.

## Conclusion

The duration of surgery, the averaged value of NR throughout surgery, and the preoperative CRP level were selected to predict early CRP changes after non-cardiac surgery under general anesthesia in patients without serious preoperative conditions or comorbidities. Predicted CRP levels on POD1 were likely associated with severity of postoperative complications. Perioperative management to suppress preoperative CRP levels, duration of surgery, and intraoperative nociceptive levels are expected to reduce major postoperative complications.

## Supporting information

S1 File (XLSX)
